# Antiandrogenic and Estrogenic Activity Evaluation of Oxygenated and Nitrated Polycyclic Aromatic Hydrocarbons Using Chemically Activated Luciferase Expression Assays

**DOI:** 10.3390/ijerph20010080

**Published:** 2022-12-21

**Authors:** Kentaro Misaki, Nguyen Minh Tue, Takeji Takamura-Enya, Hidetaka Takigami, Go Suzuki, Le Huu Tuyen, Shin Takahashi, Shinsuke Tanabe

**Affiliations:** 1 Center for Marine Environmental Studies (CMES), Ehime University, Bunkyo-cho 2-5, Matsuyama 790-8577, Japan; 2 School of Nursing, University of Shizuoka, Yada 52-1, Suruga-ku, Shizuoka 422-8526, Japan; 3 Key Laboratory of Analytical Technology for Environmental Quality and Food Safety Control (KLATEFOS), VNU University of Science, Vietnam National University, 334 Nguyen Trai, Hanoi 11400, Vietnam; 4 Department of Applied Chemistry, Kanagawa Institute of Technology, 1030 Shimo-Ogino, Atsugi 243-0292, Japan; 5 Center for Material Cycles and Waste Management Research, National Institute for Environmental Studies (NIES), Onogawa 16-2, Tsukuba 305-8506, Japan; 6 Center of Advanced Technology for the Environment, Agricultural Faculty, Ehime University, Tarumi 3-5-7, Matsuyama 790-8566, Japan

**Keywords:** polycyclic aromatic ketone, polycyclic aromatic quinone, nitrated polycyclic aromatic hydrocarbon, antiandrogenic activity, estrogenic activity, chemically activated luciferase expression assay

## Abstract

To establish the risk of the endocrine disrupting activity of polycyclic aromatic compounds, especially oxygenated and nitrated polycyclic aromatic hydrocarbons (oxy-PAHs and nitro-PAHs, respectively), antiandrogenic and estrogenic activities were determined using chemically activated luciferase expression (CALUX) assays with human osteoblast sarcoma cells. A total of 27 compounds including 9 oxy-PAHs (polycyclic aromatic ketones and quinones) and 8 nitro-PAHs was studied. The oxy-PAHs of 7*H*-benz[*de*]anthracen-7-one (BAO), 11*H*-benzo[*a*]fluoren-11-one (B[*a*]FO), 11*H*-benzo[*b*]fluoren-11-one (B[*b*]FO), and phenanthrenequinone (PhQ) exhibited significantly the potent inhibition of AR activation. All nitro-PAHs exhibited high antiandrogenic activities (especially high for 3-nitrofluoranthene (3-NFA) and 3-nitro-7*H*-benz[*de*]anthracen-7-one (3-NBAO)), and the AR inhibition was confirmed as noncompetitive for 3-NFA, 3-NBAO, and 1,3-dinitropyrene (1,3-DNPy). Antiandrogenic activity of 3-NFA demonstrated characteristically a U-shaped dose–response curve; however, the absence of fluorescence effect on the activity was confirmed. The prominent estrogenic activity dependent on dose–response curve was confirmed for 2 oxy-PAHs (i.e., B[*a*]FO and B[*b*]FO). Elucidating the role of AR and ER on the effects of polycyclic aromatic compounds (e.g., oxy- and nitro-PAHs) to endocrine dysfunctions in mammals and aquatic organisms remains a challenge.

## 1. Introduction

Environmental chemicals such as organohalogen compounds, phytoestrogens, bisphenols, phthalates, and metals have been pointed out to exhibit endocrine disrupting activity to wildlife and humans, mainly causing male reproductive system issues (e.g., cryptorchidism, hypospadias and low/decreasing semen quality) [1,2,3,4,5,6]. In particular, polycyclic aromatic hydrocarbons (PAHs) and their derivatives (e.g., oxygenated (oxy-PAHs) and nitrated (nitro-PAHs)) are found ubiquitously in diesel exhaust particles (DEP), fine particulate matter, tobacco smoke, and food and agricultural products [6,7,8,9,10,11,12,13]. These days, atmospheric pollution with high concentrations of PAHs has been reported for metropolitan areas of emerging countries [6]. It has been suggested strongly that human exposure to DEP and tobacco smoke influences the occurrence of lung cancer and cardiopulmonary diseases, due to the presence of these polycyclic aromatic compounds [6,7,8,10,11,12,13,14,15,16]. Furthermore, various developmental toxicities caused by these compounds in early and later life stages have been suggested in various epidemiological and experimental studies [15,16,17,18,19]. In humans, these include attention deficit hyperactivity disorder (ADHD), cardiovascular defects, childhood obesity, and decreased intelligence quotient (IQ) and head circumference [15,17]. In fish, these include morphological, histopathological, and neurobehavioral effects [15,16,19]. Meanwhile, endocrine disrupting effects of DEP have been reported, including reduced sperm production in adult male rodents and permanent malformations (e.g., feminization) in male offspring rodents [20]. Epidemiological studies have been focused on the relationships of air pollution with semen quality and reproductive health of young men, intrauterine growth retardation, and lower birthweight [16,21]. 

In in vitro studies using reporter gene assays, the estrogenic, antiestrogenic, and antiandrogenic activities of DEP extract samples were found to be contributed by the presence of polycyclic aromatic compounds and their metabolites depending on sample type [22,23]. The reproductive toxicity of PAHs and their metabolites is caused likely by several mechanisms [15,22,23,24,25,26,27]: (1) concentration change of steroid hormones (e.g., 17β-estradiol (E2) and testosterone) and their receptors (e.g., estrogenic (ER) and androgenic (AR) receptors); (2) direct binding interaction between a chemical and hormone receptor; and (3) signaling or signal crosstalk communication between a hormone and signaling pathway receptor (e.g., aryl hydrocarbon receptor (AhR)). Many studies evaluated mainly the hormone receptor-mediated transcriptional activities of PAHs on the basis of mechanism (2), and found the antiandrogenic activity of benzo[*k*]fluoranthene (B[*k*]FA) and benz[*a*]anthracene (B[*a*]A) [23,28,29,30,31] and the weak estrogenic activity of benzo[*a*]pyrene (B[*a*]P) and dibenzo[*a*,*h*]pyrene (DB[*a*,*h*]P) [15,30,32,33]. However, evaluation of these activities for oxy- and nitro-PAHs [31,34,35,36,37,38] is deficient. The antiandrogenic activity in several reporter gene assays of 7*H*-benz[*de*]anthracen-7-one (BAO) and 1-nitropyrene (1-NPy) has been reported [31,34,38]. An increase in the reproduction (or number of embryo) has been demonstrated in female snails exposed to BAO [39]. Meanwhile, more studies are required for determining the endocrine disrupting activities of oxy- and nitro-PAHs.

**Figure 1:**
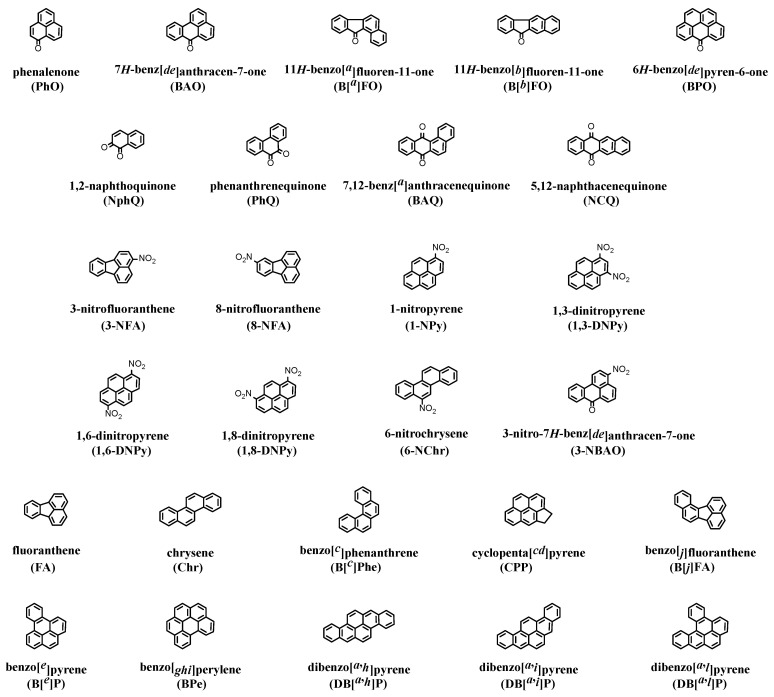
Chemical structures of the studied polycyclic aromatic compounds.

A panel of chemically activated luciferase expression (CALUX) assays with high specificity, sensitivity, and ease of handling has been developed using human osteoblastic osteosarcoma (U2OS) cells that are transfected with human steroid receptors [3,4,5,28,29,30,34,40]. Using these cells, the basic receptor activity can be measured without interferences caused by the interactions with other receptors. We have performed the toxic identification evaluation (TIE) approach for receptor activities of several environmental samples (e.g., extracts of wild animal organs and dust) with the contribution of chemicals, which were detected quantitatively in the activities of several hormone receptors using U2OS cell lines. Here, we applied the AR- and ER-CALUX assays to measure the antiandrogenic and estrogenic activities, respectively, of selected polycyclic aromatic compounds as basic data for the effect on male reproductive system. These assays were conducted to evaluate the risk of the pollutants as directly hormone-receptor-mediated gene expression through ligand binding. In the evaluation of CALUX assays with reporter gene assays, we examined whether the cytotoxicity and inherent fluorescence of a compound gave rise to an apparent receptor activity. 27 polycyclic aromatic compounds including 9 oxy-PAHs (polycyclic aromatic ketones and quinones) and 8 nitro-PAHs were evaluated (Figure 1).

## 2. Materials and Methods

### 2.1. Chemicals

The 1:1 mixture of Dulbecco’s Modified Eagle’s Medium and Ham’s F12 medium (DMEM/F12), DMEM/F12 medium (without phenol red), and fetal bovine serum (FBS) were from Life Technologies (Carlsbad, CA, USA).

Chrysene (Chr) was from Wako Pure Chemical Industries, Ltd. (Osaka, Japan). Benzo[*c*]phenanthrene (B[*c*]Phe), benzo[*j*]fluoranthene (B[*j*]FA), cyclopenta[*cd*]pyrene (CPP), dibenzo[*a*,*h*]pyrene (DB[*a*,*h*]P), dibenzo[*a*,*i*]pyrene (DB[*a*,*i*]P), dibenzo[*a*,*l*]pyrene (DB[*a*,*l*]P), 3-nitrofluoranthene (3-NFA), 1,3-dinitropyrene (1,3-DNPy), and 6-nitrochrysene (6-NChr) were from AccuStandard, Inc. (New Haven, CT, USA). 8-Nitrofluoranthene (8-NFA) was from Nihon Fine Chemical (Tokyo, Japan). All other chemicals were from Sigma–Aldrich (St. Louis, MO, USA). Benzo[*ghi*]perylene (BPe), 7,12-benz[*a*]anthracenequinone (BAQ), 1,6-dinitropyrene (1,6-DNPy), and 1,8-dinitropyrene (1,8-DNPy) were of 98% purity. Phenalenone (PhO), 1,2-naphthoquinone (NphQ), and 5,12-naphthacenequinone (NCQ) were of 97% purity. The purity of 3-NFA was >96%. The other test chemicals were of ≥99% purity. 11*H*-Benzo[*a*]fluoren-11-one (B[*a*]FO), 11*H*-benzo[*b*]fluoren-11-one (B[*b*]FO), and 6*H*-benzo[*cd*]pyren-6-one (BPO) were synthesized as described previously [14,41], and purified by column chromatography and recrystallization. The synthesized compounds were of >99% purity. The 3-nitro-7*H*-benz[*de*]anthracen-7-one (3-NBAO) was prepared by nitration of BAO [11,14]. 5α-Dihydrotestosterone (DHT; purity, >95%) and DMSO solvent (99.5%) were purchased from Wako Pure Chemical Industries, Ltd. Flutamide (99%) and E2 (≥98%) were from Sigma-Aldrich.

### 2.2. CALUX Assay 

The AR- and ER-CALUX assays for the polycyclic aromatic compounds were evaluated according to previously described methods, which used human U2OS-luc cells developed by BioDetection Systems b.v. (Amsterdam, The Netherlands) [40]. The cells are transfected stably with human AR and ER, respectively, and a luciferase reporter gene under their respective steroid receptor response elements [3,4,5,28,29,30,34,40,42,43]. Cells were maintained in a DMEM/F12 medium that was supplemented with 7.5% FBS. They were incubated at 37 °C in a humidified atmosphere containing 5% carbon dioxide. They were then plated for 1 day on 96-well microplates, and exposed for 24 h to each compound in DMEM/F12 medium (without phenol red) that was supplemented with 4.9% dextran-coated charcoal-treated FBS. For measurement of AR antagonistic activity, the cells were exposed to each compound in the presence of a half-maximum effective concentration (EC_50_) level (100 pM) of dihydrotestosterone (DHT). The medium was then removed, and the cells were lysed. A solution of luciferin was then added, and the luminescence intensity was measured with an ARVO™ X3 luminometer from PerkinElmer (Waltham, MA, USA). A sigmoid dose–response curve for a chemical was fitted to the Hill equation given by Equation (1) using SigmaPlot 11.0 from Systat Software Inc. (San Jose, CA, USA).

y = *min* + (*max* − *min*)/(1 + (*a*_1/_x)*^a^*^2^) (1)

where, y and x are the measured luciferase induction level and compound concentration, respectively. The value of *min* corresponds to the luciferase induction level at maximum inhibition by the reference antagonist (≈0% for flutamide) in the antiandrogenic assay and at the minimum induction by the DMSO control (≈0%) in the estrogenic assay. The value of *max* corresponds to luciferase induction level with the positive control, i.e., 100 pM DHT in antiandrogenic assay and 1 nM E2 in estrogenic assay (≈100%). *a*_1_ is half-maximum inhibitory concentration (IC_50_) in AR-CALUX and EC_50_ in ER-CALUX. *a*_2_ is the curve slope. When maximum inhibition could not be achieved because of cytotoxicity, low solubility or unavailability of higher concentrations, the dose–response curve was fitted to Equation (1) with a *min* value of 0. The REP values in mol base were obtained by dividing the ICs or ECs of the standard by the ICs or ECs of target compound, respectively. 

All measurements were conducted in triplicate wells. They were repeated twice and trice in the AR- and ER-CALUX assays, respectively, for each concentration in dose–response curve. The calculated AR-CALUX assay IC_50_ of flutamide as the AR antagonist standard at the EC_50_ DHT level was 260 ± 20 nM (mean ± SD; *n* = 4), which was comparable to our previous study [3,4,5]. Cell viability was confirmed using 3-(4,5-dimethylthiazol-2-yl)-2,5-diphenyltetrazolium bromide (MTT) assay [3,4]. If the cell viability was <80% in the positive control, the corresponding exposure dose was considered cytotoxic and excluded from the calculation. Cytotoxicity was confirmed for phenanthrenequinone (PhQ) and 3-NBAO at a concentration of 1 μM. Competitive inhibition for AR ligand binding-mediated activation was confirmed with the absence of inhibition by coexposure to the reference agonist DHT at high concentration (100 × EC_50_ level, 10 nM) [3,4,40].

## 3. Results

### 3.1. AR-CALUX Assay for Antiandrogenic Activity

**Figure 2:**
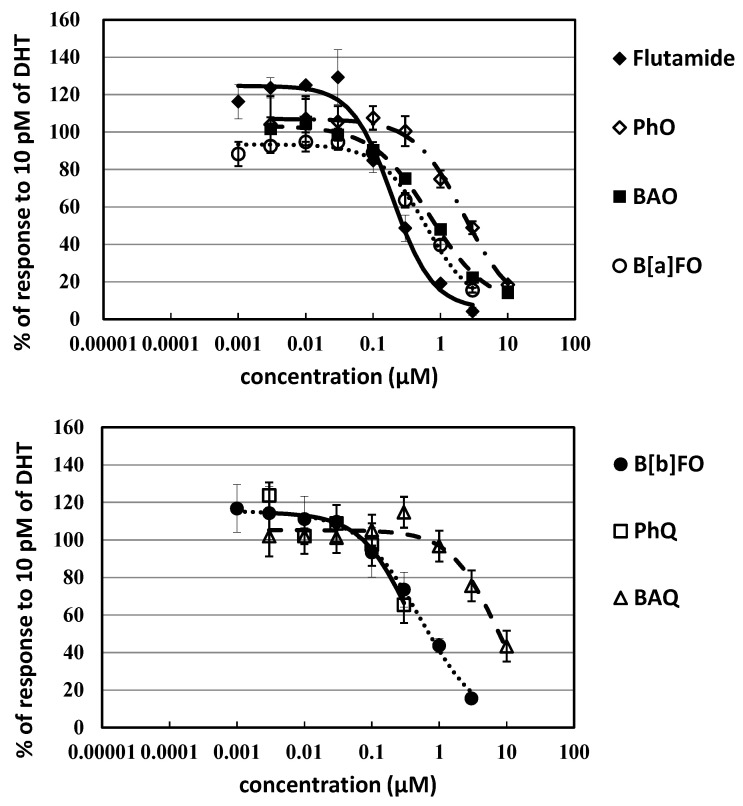
Dose–response curves of antiandrogenic activities for oxy-PAHs in 24 h exposure experiments with the AR-CALUX assay.

Among oxy-PAHs, the 4-ring compounds BAO, B[*a*]FO, and B[*b*]FO exhibited significant inhibition of AR activation (antiandrogenic activity), which was comparable with flutamide with the molar IC_50_-based relative potency (REP_50_) values of 0.37, 0.46, and 0.43, respectively (Figure 2 and Table 1). The 3-ring PhQ showed a relatively strong potency with a molar REP_50_ of 0.59, and its cytotoxicity was confirmed at 1 μM. The potencies of 3-ring PhO and BAQ were also significant, but one order of magnitude lower than that of flutamide, with REP values of 0.11 and 0.049, respectively. Antiandrogenic activity was not observed for the 2-ring NphQ, 4-ring NCQ, and 5-ring BPO (Figure 2).

**Figure 3:**
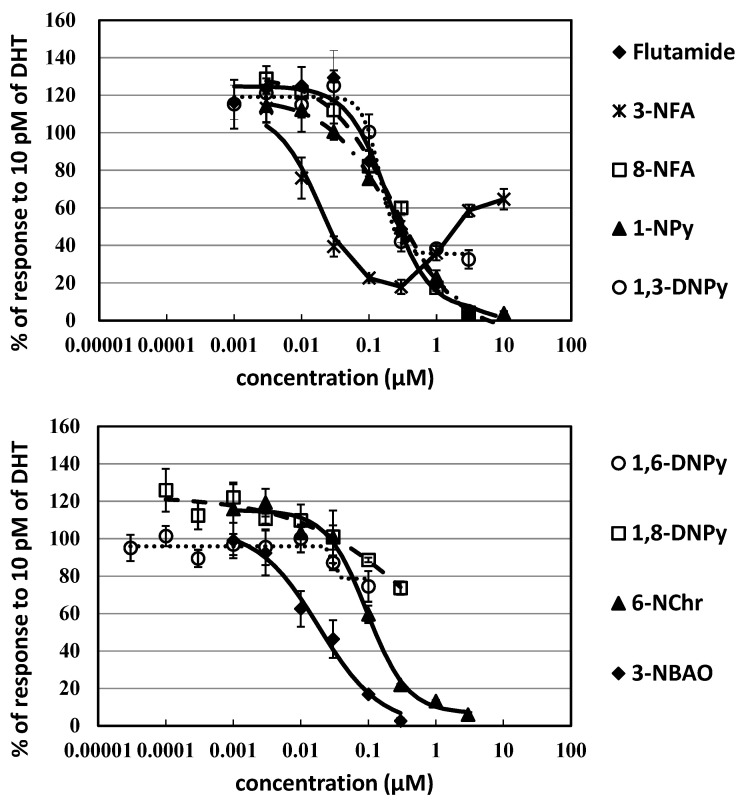
Dose–response curves of antiandrogenic activities for nitro-PAHs in 24 h exposure experiments with the AR-CALUX assay.

The antiandrogenic activity was very high for the tested nitro-PAHs (Figure 3 and Table 1). Characteristically, the dose–response curve was U-shaped for the antiandrogenic activity of the 4-ring 3-NFA. That is to say, AR-mediated luciferase activity was inhibited at low concentrations and the inhibition was attenuated at concentrations >300 nM by 3-NFA. For 1,3-DNPy, the maximum inhibition of AR-mediated luciferase activity corresponded to about 30% of the activity induced by the DHT control. To compare appropriately the antiandrogenic activities among compounds at low concentrations, the IC and REP values of 3-NFA and 1,3-DNPy were obtained by fitting the dose–response curve to the Hill equation with response data points at low concentrations and with *min* set as 0 (additionally with *max* set as 100 for 3-NFA) (Figure 3). The highest flutamide-REP was confirmed for 3-NFA (molar REP_50_ = 21). The REP was especially high for 3-NBAO (15) though the cytotoxicity was observed at 1 μM. The 4-ring 6-NChr showed a strong potency with REP of 3.3. Meanwhile, the 4-ring 8-NFA, 1-NPy, and 1,3-DNPy also exhibited relatively strong potencies with REP values of 0.91, 1.1, and 1.3, respectively. The 4-ring 1,8-DNPy showed a significant potency with a REP of 0.39. We were unable to determine the REP for 1,6-DNPy due to insufficient inhibition at the maximum soluble concentration. Nevertheless, a >20% inhibition of AR activation was confirmed at 100 nM 1,6-DNPy (Figure 3). The androgenic activity was examined for 3-NFA only in order to confirm the possibility that the remarkable attenuation of AR activation for 3-NFA is derived from the androgenic activity of 3-NFA.

**Figure 4:**
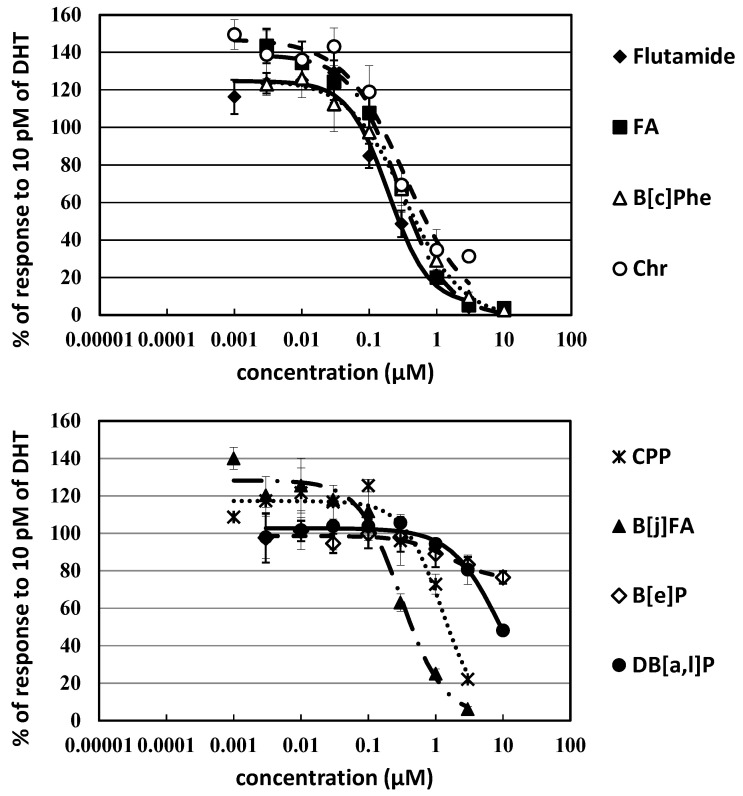
Dose–response curves of antiandrogenic activities for PAHs in 24 h exposure experiments with the AR-CALUX assay.

Among PAHs examined in the present study, relatively strong antiandrogenic potency were observed for the 4-ring compounds fluoranthene (FA) and Chr, and the 5-ring B[*j*]FA with flutamide-relative REP_50_ values of 1.1, 0.99, and 0.81, respectively (Figure 4 and Table 1). The REP = 0.62 found for B[*c*]Phe was significant. The 5-ring CPP and 6-ring DB[*a,l*]P exhibited significant potencies, but one order of magnitude less potent than flutamide (REP_50_ = 0.16 and 0.030, respectively). Antiandrogenic activity was not observed for the 6-ring BPe, DB[*a*,*h*]P, and DB[*a*,*i*]P. We were unable to determine the REP of benzo[*e*]pyrene (B[*e*]P), due to insufficient inhibition at the maximum soluble concentration (>15% inhibition at 10 μM) (Figure 4). 

### 3.2. ER-CALUX Assay for Estrogenic Activity

**Figure 5:**
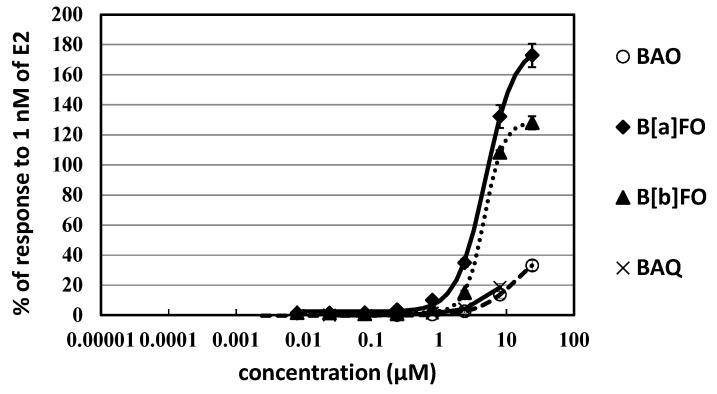
Dose–response curves of estrogenic activities for oxy-PAHs in 24 h exposure experiments with the ER-CALUX assay.

In the ER-CALUX assay, E2 was used as an ER agonist standard with an EC_50_ of 10 pM [3,5]. Significant potencies in ER activation dependent on dose–response curve were confirmed for two polycyclic aromatic ketones, both B[*a*]FO and B[*b*]FO, with molar EC_50_-based REP relative to E2 of 2.1 × 10^−6^ approximately 3 times higher than BAO (Figure 5 and Table 1). An achievement to 10% level of the response to a high E2 dose (100 × EC_50_ level, 1 nM) exposure was confirmed for BAQ in other oxy-PAHs, 1-NPy in nitro-PAHs and B[*c*]Phe, Chr, B[*j*]FA, and DB[*a*,*i*]P in PAHs. Meanwhile, most compounds showed no or very low ER activities under the 10% response of 1 nM E2 (Table 1). 

## 4. Discussion

The results of antiandrogenic and estrogenic activity obtained in the present study were evaluated with the consideration of contributing factors and the comparison of the activity with one by other researchers (Table 1) [22,28,29,30,31,33,34,36,38,44].

### 4.1. Antiandrogenic Activity in the CALUX Assay

The antiandrogenic activity was confirmed for 21 polycyclic aromatic compounds; 6 oxy-PAHs, 8 nitro-PAHs, and 7 PAHs among the 27 compounds examined with the AR-CALUX assay (Figure 2, Figure 3 and Figure 4 and Table 1). The concentrations were in the range where the effect of cytotoxicity was excluded because cytotoxicity affects apparent variation of antiandrogenic activity [45,46]. In the coexposure to DHT at 10 nM (100 × EC_50_), inhibition of AR-mediated luciferase activity was observed for 3-NFA (26% and 82% inhibition at 100 nM and 10 μM, respectively), 3-NBAO (29% and 60% inhibition at 30 nM and 300 nM, respectively), and 1,3-DNPy (33% at 3 μM) (details not shown). Therefore, these nitro-PAHs were recognized as noncompetitive inhibitors for AR activation. The AR inhibition with a noncompetitive fashion was previously reported for several compounds such as bisphenol A, tris(1,3-dichloro-2-propyl) phosphate (TDCIPP), and harmine hydrochloride [47,48,49].

A specific attenuation of inhibition of AR-mediated luciferase activity was observed at concentrations of >300 nM for 3-NFA (Figure 3). Weak androgenic activity was observed for 3-NFA at concentrations of ≥1 μM, but the maximum induction was below 8% level of maximum response of DHT exposure (10 nM) (16% of the EC_50_ level of DHT) (data not shown). The androgenic activity was not enough to explain the attenuation of antiandrogenic activity by approximately 40% at high concentrations compared to at 100 nM. 

**Table 1:** Antiandrogenic and estrogenic activities and REP values for the studied oxy-PAHs, nitro-PAHs, and PAHs.

	Antiandrogenic Activity										Estrogenic Activity							
	Present Study					Previous Study					Present Study				Previous Study			
	IC_25_ (μM)	REP_mol_ (IC_25_)	IC_50_ (μM)	REP_mol_ (IC_50_)		REP_mol_	IC_25_ (μM)				EC_50_ (μM)	REP_mol_ (EC_50_)			REP_mol_	REP_mol_	REP_mol_	REP_mol_
**Compound *^a^***						**U2OS**	**CHO*^g^***	**PC3 *^h^***	**Yeast *^i^***						**U2OS *^e^***	**T47D *^l^***	**VM7 *^n^***	**MVLN *^o^***
**Standard**																		
**Flutamide**	**0.11**	**1**	**0.26**	**1**		**1**												
**E2**											**1 × 10^−5^**	**1**			**1**	**1**	**1**	**1**
**Oxy-PAHs**																		
**PhO**	**0.84**	**0.12**	**2.0**	**0.11**							**U *^j^***	**U**						
**BAO**	**0.20**	**0.64**	**0.71**	**0.37**		**0.014 *^c^***					**16**	**7.5 × 10^−7^**						**3.0 × 10^−6^**
**B[*a*]FO**	**0.30**	**0.43**	**0.67**	**0.46**							**4.8**	**2.1 × 10^−6^**						
**B[*b*]FO**	**0.15**	**0.81**	**0.53**	**0.43**							**4.8**	**2.1 × 10^−6^**						
**BPO**	**nd *^b^***	**nd**	**nd**	**nd**							**U**	**U**						
**NphQ**	**nd**	**nd**	**nd**	**nd**							**nd**	**nd**						
**PhQ**	**0.18**	**0.63**	**0.39**	**0.59**							**nd**	**nd**						
**BAQ**	**2.5**	**0.056**	**6.4**	**0.049**							**18% activation at 8 μM**							**1.4 × 10^−6^**
**NCQ**	**nd**	**nd**	**nd**	**nd**							**U**	**U**						
**Nitro-PAHs**																		
**3-NFA**	**0.0053**	**32**	**0.014**	**21**					**+**		**U**	**U**						
	**increase at > 0.3 μM**																	
**8-NFA**	**0.098**	**1.3**	**0.27**	**0.91**							**nd**	**nd**						
**1-NPy**	**0.094**	**1.4**	**0.22**	**1.1**			**0.3 < IC_25_ ≤** **1**		**+**		**14% activation at 10 μM**							
**1,3-DNPy**	**0.13**	**0.88**	**0.21**	**1.3**							**U**	**U**						
**1,6-DNPy**	**>20% inhibition at 0.1 μM**								**+**		**U**	**U**						
**1,8-DNPy**	**0.17**	**0.91**	**0.72**	**0.39**							**U**	**U**						
**6-NChr**	**0.039**	**3.2**	**0.088**	**3.3**							**NA *^k^***	**NA**						
**3-NBAO**	**0.0051**	**23**	**0.018**	**15**					**+**		**NA**	**NA**						
**PAHs**																		
**Ant**						**0.2 *^d^***	**3 < IC_25_ ≤** **10**										**8.3 × 10^−7^**	
**Phe**						**0.1 *^e^***	**nd**								**<3 × 10^−9^**		**1.2 × 10^−6^**	
**FA**	**0.14**	**1.0**	**0.30**	**1.1**		**0.96 *^f^***	**1 < IC_25_ ≤** **3**									**ND *^m^***	**4.1 × 10^−7^**	
**Py**						**0.41 *^f^*, 0.03 *^e^***	**3 < IC_25_ ≤** **10**	**+**								**5.3 × 10^−7^**	**nd**	
**B[*a*]A**						**1.3 *^f^,* 0.4 *^d^***	**1 < IC_25_ ≤** **3**	**+**							**<2 × 10^−9^**	**1.6 × 10^−6^**		**7.9 × 10^−7^**
**B[*c*]Phe**	**0.11**	**1.0**	**0.37**	**0.62**							**27% activation at 8 μM**							
**Chr**	**0.084**	**1.8**	**0.33**	**0.99**		**nd *^f^***	**3 < IC_25_ ≤** **10**	**+**			**15% activation at 8 μM**					**U**	**nd**	
**B[*b*]FA**							**nd**	**+**										
**B[*k*]FA**						**2.6 *^f^***	**0.3 < IC_25_ ≤** **1**	**+**								**nd**		
**B[*j*]FA**	**0.13**	**0.84**	**0.29**	**0.81**			**1 < IC_25_ ≤** **3**	**+**			**13% activation at 3 μM**							
**CPP**	**0.58**	**0.14**	**1.3**	**0.16**							**nd**	**nd**						
**B[*a*]P**						**0.4 *^d^*, 0.1 *^e^***	**3 < IC_25_ ≤** **10**	**+**							**2 × 10^−6^**	**3.1 × 10^−6^**	**5.3 × 10^−6^**	**2.6 × 10^−7^**
**B[*e*]P**	**>15% inhibition at 10 μM**							**+**			**U**	**U**						
**DB[*a*,*h*]A**						**nd *^d^***												
**IdP**						**0.35 *^f^***		**+**										
**BPe**	**nd**	**nd**	**nd**	**nd**		**nd *^f^***		**+**			**nd**	**nd**					**nd**	
**DB[*a*,*h*]P**	**nd**	**nd**	**nd**	**nd**		**<0.003 *^e^***					**nd**	**nd**			**2 × 10^−5^**			
**DB[*a*,*i*]P**	**nd**	**nd**	**nd**	**nd**							**14% activation at 3 μM**							
**DB[*a*,*l*]P**	**3.6**	**0.037**	**8.9**	**0.030**							**U**	**U**						

*^a^*Ant, abthracene; Phe, phenanthrene; Py, pyrene; B[*a*]A, benz[*a*]anthracene; benzo[*b*]fluoranthene; B[*b*]FA; benzo[*k*]fluoranthene; B[*k*]FA;benzo[*a*]pyrene; B[*a*]P; DB[*a*,*h*]A, dibenz[*a*,*h*]anthracene; IdP: indeno[1,2,3-*cd*]pyrene. *^b^* not detected. *^c^* Previously reported by Weiss et al. [34]. *^d^* Previously reported by Marin-Kuan et al. [29]. *^e^* Previously reported by Pieterse et al. [30]. *^f^* Previously reported by Alvarez-Muñoz et al. [28]. *^g^* Previously reported with Chinese hamster ovary (CHO) cells by Vinggaard et al. [31]. *^h^* Previously reported with human prostate cancer (PC3) cells by Okamura et al. [22]. +, positive. *^i^* Previously reported with yeast by Otsuki et al. [38]. +, positive. *^j^* < 10% response of 1 nM E2. *^k^* not available. *^l^* Previously reported with human breast cancer (T47D) cells by Šimečková et al. [44]. *^m^* Not determined. *^n^* Previously reported with human breast cancer (VM7) by Boonen et al. [33]. *^o^* Previously reported with human breast cancer (MVLN) cells by Machala et al. [36].

The fluorescence emitted from several nitro-PAHs (e.g., 3-NFA) absorbed into cells was confirmed to have no effect on the luminescence intensity as relative light units (RLU) for AR-activated luciferase expression in previous report [50,51] and our fluorescence measurements with an FP8300 specrtometer from JASCO (Tokyo, Japan) (data not shown). Therefore, it is suggested that the U-shaped curve in the CALUX assay may be derived from the alteration of noncompetitive action mode in AR inhibiton by 3-NFA dependent on its concentration.

AR antagonistic activities have been reported for several polycyclic aromatic compounds including mainly PAHs in CALUX assays with U2OS, human prostate cancer cells, etc. (Table 1) [28,29,30,31,34,35,44]. The REP value of FA is similar to the value observed in the previous study with U2OS cells (Figure 4) [28,44]. Here, the antiandrogenic activity of 3 PAHs (B[*c*]Phe, CPP, and DB[*a*,*l*]P) was confirmed for the first time. There is presumably the tendency that the 4- and 5-ring PAHs with appropriate shapes similar to endogeneous androgens for AR ligand binding pocket (i.e., FA, B[*a*]A, B[*c*]Phe, Chr, B[*k*]FA, B[*j*]FA, and B[*a*]P) have strong AR antagonistic activities that are comparable with flutamide [52].

Meanwhile, dibenz[*a*,*h*]anthracene (DB[*a*,*h*]A), BPe, DB[*a*,*h*]P, and DB[*a*,*i*]P are inactive. B[*e*]P was shown to be a partial antagonist because of the dose–response curve. AhR-mediated antiandrogenic activity was proposed in prostate cancer cells [22,25,27,53,54]. In reporter gene assays using prostate cancer cells, significant correlations between antiandrogenic and AhR agonistic activities have been demonstrated by several PAHs at 100 nM [22]. U2OS (osteoblast sarcoma) cells have low expression levels of AhR and CYPs. A small effect of AhR on AR inhibition activity (the metabolites of PAHs with CYPs and binding interaction between AhR and AR) is predicted [55]. For U2OS cells, the effect of AhR on ERα-activation has been reported: The change of expression amount in ERα-mediated activation was not caused by AhR ligand (i.e., dioxin) in CALUX assay with mammalian ERα-transfected U2OS-luc cells, if AhR was not transfected in these cells [56]. Therefore, we believe that our assay results are reflected from AR ligand binding itself.

Among oxy-PAHs, BAO exhibited significant antiandrogenic activity; however, the potency was about one order of magnitude lower than flutamide (Table 1, Figure 2). The antiandrogenic activity of PhO, B[*a*]FO, B[*b*]FO, PhQ, and BAQ was confirmed for the first time. There was the tendency that the antiandrogenic activity of oxy-PAHs was slightly lower than PAHs. This was caused likely by the ease of metabolism though the metabolism speed may be significantly slower in U2OS cells than male reproductive cells [52].

The antiandrogenic activity was considerably high for the nitro-PAHs (Table 1 and Figure 3). It is characteristic of 3-NFA that AR activation was inhibited at low concentrations but the inhibition was attenuated at concentrations of >300 nM (U-shaped curve). The antiandrogenic activities of 3-NFA and 3-NBAO, which is known a strong direct-mutagen [11], at low concentrations were especially high among the studied polycyclic aromatic compounds. Nowadays, especially high thyroid receptor (TR) α activation was reported for 3-NBAO, with REP_50_ to triiodothyronine (T3) of 0.11 [23], therefore the influence of 3-NBAO on endocrine disruption is to be further evaluated at low concentrations. The high antiandrogenic activity of 1-NPy has been reported with Chinese hamster ovary (CHO K1) cells transfected with human AR by Vinggaard et al. [31]. High antiandrogenic activity has been reported with yeast two-hybrid assay transfected with human AR for nitro-PAHs (e.g., 3-NBAO, 3-NFA, and 1,6-DNPy) [38]. In the present study, AR-mediated luciferase activity was inhibited by 3-NFA, 3-NBAO, and 1,3-DNPy in noncompetitive rather than competitive fashion. The antiandrogenic activities of several nitro-PAHs are presumably due to their appropriate size and shape and the electrostatic stability of nitro function via hydrogen bond in an AR ligand binding pocket [37,52,57,58]. There is a possibly of higher antiandrogenic activities with their nitroso and amino metabolites. Further studies are necessary to understand the mechanism of the high antiandrogenic activities in the studied cells including U2OS cells.

### 4.2. Estrogenic Activity in the CALUX Assay

Among polycyclic aromatic compounds examined in the present study, significant potency in ER activation was confirmed for B[*a*]FO and B[*b*]FO, and this potency is comparable to B[*a*]P previously reported with U2OS-luc cells (Figure 5, Table 1). Higher ER activity of DB[*a*,*h*]P than B[*a*]P has been reported with U2OS-luc cells; however, this activity was not confirmed in the present study. The ER activation of BAO was also observed with approximately three times lower than B[a]FO and B[b]FO. 18% level of the response of E2 exposure (1 nM) was confirmed for BAQ at 8 μM (Figure 5, Table 1). In human breast cancer cells transfected with human ER, ER activities that were higher than B[*a*]P were confirmed for B[*a*]A, BAO, BAQ, and anthraquinone (AQ) [36]. In yeast two-hybrid assay transfected with human ER, the high estrogenic activity was not confirmed for BAO, B[*a*]FO, B[*b*]FO, AQ, and BAQ, but for 1,2-chrysenequinone and 2 benzo[*a*]pyrenequinones [37]. Significant potency has been reported for 4- and 5-ring oxy-PAHs, and this is derived probably from their appropriate size and shape and the electrostatic stability of carbonyl function via hydrogen bonding in an ER ligand binding pocket [37,57,58]. Further studies are necessary to understand the mechanism of the estrogenic activities of the studied compounds in the considered cells. High estrogenic activities have been reported for several hydroxy-PAHs (REP values in the range of 1.1 × 10^−6^–2.2 × 10^−4^) with human breast cancer cells [32]. Superior ER activities have been confirmed for the polar fractions of atmospheric pollutant and tobacco extracts that contain hydroxy-PAHs to semipolar fractions that contain polycyclic aromatic ketones and quinones [59,60,61]. The results of estrogenic activity in the present study reflect the significant contribution of oxy-PAHs such as ketones and quinones in these pollutants though the contribution of hydroxy-PAHs is expected to be particularly high.

### 4.3. Endocrinological Risk Implications of Oxy-PAHs and Nitro-PAHs

Epidemiological studies have been reported mainly on the relationships of air pollution with semen quality and reproductive health of young men, intrauterine growth retardation, and lower birthweight [16,21,62,63,64,65], therefore the targets of the present study are antiandrogenic activities and estrogenic activities of polycyclic aromatic compounds, especially oxy-PAHs and nitro-PAHs in CALUX assays. Estrogenic and antiandrogenic activity have been presented for atmospheric extract samples and the contribution of PAHs and their semipolar or polar derivatives is suggested in several studies [22,59,60]. 

Endocrine disrupting effects (e.g., reduction of sperm production and deterioration of sperm ability) of DEP on male rodents have been reported. In addition, antiandrogenic activity has been suggested [20,66]. Moreover, the effects of several PAHs on the reproductive system have been reported [67,68,69,70,71,72,73,74]. For example, B[*a*]P was found to reduce testosterone levels in rat testicular cells by the direct disturbance of steroidogenesis [67]. It was also presented that maternal exposure to B[*b*]FA disturbed normal sperm function in male offspring mice [68]. Moreover, long-term exposure to environmental levels of Phe in male mice induced the disruption of spermatogenesis, and changes in testicular AR and ERα, and serum E2 levels were observed [69]. Meanwhile, estrogenic effects (e.g., increase of uterine weight and hypertrophy of luminal epithelium via ERα) of B[*a*]A, B[*a*]P, and FA were observed in immature female rats [70]. In aquatic environments, pollution by several polycyclic aromatic compounds has also been reported [8,15,16,28,39,60,75], and the effects of these compounds on aquatic organisms are growing concerns [39,71,72,73,74,75]. AR is a nuclear receptor which when bound to androgenic testosterones (e.g., DHT and 11-ketotestosterone in mammals and fish, respectively) alters gene transcription in favor of more masculine phenotypic characteristics. The alternations are generally reversible in adults, and failure to regulate properly this pathway during periods of embryonic development may result in permanent malformations (e.g., feminization of male offspring) [71]. For example, Phe has been shown to decrease the levels of spermatozoa and mature oocyte in male and female fish, respectively, due to a decrease in the levels of sex steroid hormones in the brain-pituitary-gonadal (BPG) axis [72,73]. An increase in reproduction (the number of embryo) has been observed in female snails exposed to BAO [39]. However, the target compounds examined for endocrine disrupting activities in mammals and aquatic organisms have been limited [39,67,68,69,70,71,72,73,74,75]; therefore, further studies are required for polycyclic aromatic compounds, especially oxy- and nitro-PAHs with antiandrogenic and estrogenic activities in the present CALUX assay results. In addition, low-dose and nonmonotonic dose–response curves of compounds in humans and animals may have unclear mechanisms that are associated with AR inhibition and ER activation of the compounds [76]. In the present study, antiandrogenic activity was observed for 3-NFA with a U-shaped dose–response curve. Meanwhile, the percentage of sperm production in male fish of *Sebastiscus marmoratus* exposed to Phe has been shown to have a U-shaped dose–response curve, and this phenomenon was believed to be a result of biotransformation enzyme activity alteration in the fish brain [73].

Various developmental toxicities in early and later life stages, which are caused by atmospheric pollutant extracts and polycyclic aromatic compounds have been suggested [15,16,17,18,42,74,77,78,79,80]. These include childhood obesity, cardiovascular defects, ADHD, and decreased IQ and head circumference in humans [15,17,77]. Kamelia et al. have reported a high correlation of developmental toxicity of petroleum substance extracts in mouse embryonic stem cell test (EST) with AhR agonistic activity (determination coefficient: R^2^ = 0.80). A correlation was not found with AR antagonistic activity [42]. Developmental toxicities in fish have been examined for a series of polycyclic aromatic compounds [16,19,74,78,79,80]. Knecht et al. showed the relationship of developmental toxicity (morphological, histopathological, and neurobehavioral effects) in zebrafish embryo with several mechanisms such as AhR signaling and oxidative stress with reactive oxygen species (ROS) for oxy- and nitro-PAHs [19,78,79,80]. The AhR activities of the oxy-PAHs (i.e., B[*a*]FO, B[*b*]FO, and NCQ) were found significant in our previous study [41,81]. Several nitro-PAHs such as 3-NBAO, dinitropyrenes, and 3-NFA, which are known as ROS and AhR-mediated CYPs inducers, cause embryonic morphological and behavioral effects [78,80]. The relationships of estrogenic, antiestrogenic, and antiandrogenic activity of PAHs with endogenous AhR have been reported for several cells and reporter gene assays [6,15,22,23,25,27,33,43,53,54,56,67,68,70,71]. However, the effect of AhR on the AR inhibition and ER activation (the metabolites of compounds with CYPs and binding interaction between AhR and AR or ER) is predicted to be small in the present study with U2OS-luc cells [40,55,56].

Several quantitative structure-activity relationship (QSAR) modeling methods, which are based on binding affinity and transcription activities of polycyclic aromatic compounds with sex hormone receptors, have been developed for ligand binding domains in sex hormone receptors [37,52,57,58]. The presented data on the AR antagonistic and ER agonistic activities might help in exploiting more appropriate QSAR modeling for these compounds, and predicting the activities of unknown compounds. AR noncompetitive inhibition, not via AR ligand binding domain was also exhibited for several nitro-PAHs. The contribution of PAHs and nitro-PAHs in antiandrogenic activities and contribution of oxy-PAHs in estrogenic activities are strongly predicted based on the presented assay results and previously reported analytical data on the extracts of atmospheric pollutants [6,7,8,9,10,11,12,13,82,83]. It has been found that majority of the contributions in these activities were not explained by known compounds found in the extract samples of atmospheric pollutants. Therefore, it is urgent to further explore other potentially unknown AR antagonists and ER agonists in atmospheric pollutants.

## 5. Conclusions

27 polycyclic aromatic compounds were examined with an AR-CALUX assay, which is unaffected by the cytotoxicity and fluorescence derived from the studied compounds. The antiandrogenic activity of 6 oxy-PAHs (e.g., B[*a*]FO, B[*b*]FO, and PhQ), 8 nitro-PAHs (notably high for 3-NFA and 3-NBAO), and 7 PAHs were confirmed. The prominent estrogenic activity that was dependent on dose–response curve was confirmed for 2 oxy-PAHs (i.e., B[*a*]FO and B[*b*]FO). A 10% level of positive control maximum response was also achieved for other 6 compounds. Elucidating the roles of AR and ER on the effects of polycyclic aromatic compounds including oxy-PAHs and nitro-PAHs to endocrine dysfunctions in mammals and aquatic organisms is still a challenge.

## Data Availability

Not applicable.
